# Impact of the COVID-19 on foreign direct investment inflows in emerging economies: evidence from panel quantile regression

**DOI:** 10.1186/s43093-022-00133-9

**Published:** 2022-07-28

**Authors:** Sinem Koçak, Özge Barış-Tüzemen

**Affiliations:** Department of Econometrics, Graduate School of Social Science, Karadeniz Technical University, 61000 Trabzon, Turkey

**Keywords:** COVID-19 pandemic, FDI, Emerging countries, Panel quantile regression

## Abstract

The study explores the role of the COVID-19 pandemic on foreign direct investment in 12 emerging countries for the period between 2014 and 2021. The world pandemic uncertainty index is used, and panel quantile regression approach is employed to analyze the effect of the pandemic on foreign investment inflows. Unlike the conditional mean regression analysis, panel quantile regression gauges the independent variables at the different locations of the dependent variable. For this reason, it provides a more comprehensive illustration of the impact of the independent variables on dependent variables. The results show that the pandemic has an inverse effect on foreign direct investment in low- and middle-foreign investment receiving countries, while the effect is insignificant in high-foreign investment receiving countries. Apparently, the health crisis has been further harmful to the countries that have weaker economic structures.

## Introduction

From the beginning of the 1980s, worldwide capital movements have been liberalized. Foreign direct investment (FDI, hereafter) has grown and become more competitive [42]. The locomotive role of the FDI on economic growth and development has been controversial. It has been widely accepted that FDI has a range of benefits to the host country's economy. It, for example, brings the standard of living and prospects for economic growth, accelerates technology, enhances managerial ability, and enlarges their export markets, and those advantages outweigh its disadvantages [34, 62]. FDI involves long-term and permanent mercantile commitments, and for this reason, it differs from the other types of capital flows such as portfolio investment. Due to this feature, FDI encourages investors to assume a more proactive role in the decision-making process and might help the firms to restructure [14]. Some other studies, meanwhile, emphasize the risk of FDI in receiving countries. As an example, FDI might devastate local abilities. Another possible risk is natural resources extraction without sufficiently compensating poor countries. Thus, one would say the impacts of FDI rely on the type of FDI, firm characteristics, economic circumstances, and policies [45].

In late December 2019, a new disease caused by severe acute respiratory syndrome coronavirus 2 (SARS-CoV-2) was defined and has been officially named COVID-19. Afterward, COVID-19 had been spread to other countries from its originated country and was called a pandemic by the World Health Organization (WHO) on March 11, 2020. “Pandemics are large-scale outbreaks of infectious diseases that can greatly increase morbidity and mortality over a wide geographic area and cause significant economic, social, and political disruption” [40]. COVID-19 has seriously hit the entire world economically, socially, and psychologically. The detrimental impact of COVID-19 on the worldwide economy is inescapable. The direct economic effects of the pandemic can be summarized as income declined due to unemployment and the loss of working hours. As a result of those, total aggregate demand had dropped, thus lower demand led to output loss [49]. Also, COVID-19 lifted the export and import expenses, hence it diminished international trade and caused a lack of efficiency. Apart from these, the tourism industry, which is the main income source for many countries has been severely damaged by the pandemic [41].

In this study, the effects of COVID-19 on FDI in emerging countries have been investigated. Emerging countries vary from list to list, but in the study, the most recognized 12 emerging countries are used and 5 of those countries are among the top 20 FDI host economies.^1^ The data set covers the period between 2014Q1 and 2021Q3, and the availability of data was determinative in the selection of countries. After the COVID-19 outbreak, little inquiry has been done to be able to see its effect on FDI. Therefore, this study is expected to fill the gap in the literature. Besides, the most novel point that distinguishes this study from the previous ones is to examine the role of the pandemic on FDI within the concept of the world pandemic uncertainty index. This study also contributes to the literature by using panel quantile regression. “Quantile regression models allow the researcher to account for unobserved heterogeneity and heterogeneous covariates effects, while the availability of panel data potentially allows the researcher to include fixed effects to control for some unobserved covariates” [20]. Besides, quantile regression provides a more extensive analysis by showing different effects of the independent variables on dependent variables. Three quantiles have been used in the study, which are the 25th, 50th, and 75th quantiles. The impact of pandemic uncertainty index on foreign direct investment is negative and heterogeneous at all quantiles and becomes significant at the low quantile (0.25th quantile) and the middle quantile (0.50th quantile), implying that the influence of the COVID-19 pandemic on FDI is detrimental, and the effects are more significant for the low-FDI countries and the middle-FDI countries than the high quantile. The coefficient is negative but insignificant at the 0.75th quantile.

The remainder of the paper is arranged as follows. The FDI flow trend amid COVID-19 is presented in “Global FDI and Covid-19” section, while “Related literature” section considers the literature review. The data set and econometrics methodology are given in “Data set and methodology” section, followed by “Results and discussion” section offering empirical findings and discussions. Finally, the conclusion and policy implications are provided in “Conclusions” section.

## Global FDI and COVID-19

In order to prevent the spreading of the virus, governments in many countries had implemented several health restrictions such as shutdowns, travel restrictions, and national security-related inspection of investments. International trade has been heavily affected by all those pandemic measures [44, 46]. In addition, another macroeconomic variable FDI has also been adversely impacted by the pandemic as well. In 2020, global FDI flows dropped by one in three to $1 trillion which is the lowest point after the global financial crisis. The highest productive sorts of investment such as greenfield investment in industrial and infrastructure projects, especially in developing countries, have been damaged by COVID-19. This implies that international production, the engine of global economic growth and development, has been severely influenced [59].

**Fig. 1:**
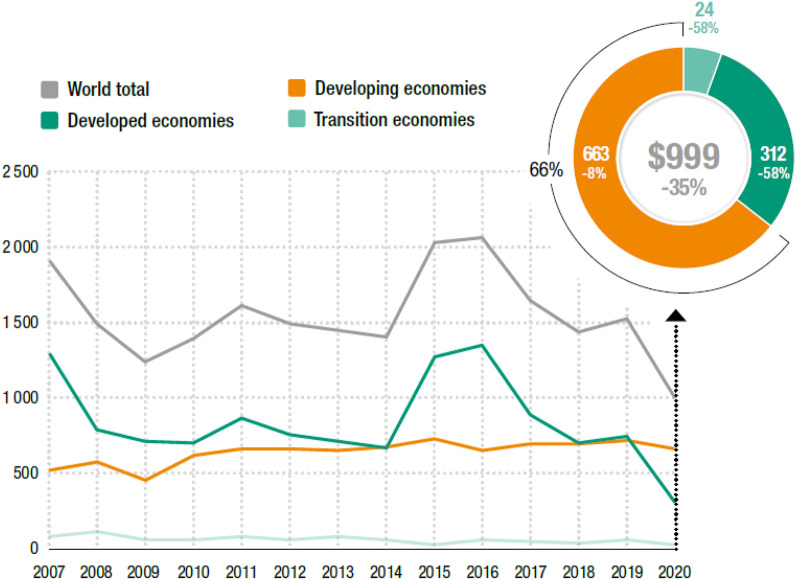
FDI inflows selected economies (billion dollars). *Source*: UNCTAD

The devastating effects of the pandemic on the FDI flow trend differ by the group of developing and developed economies. Figure 1 presents FDI inflows between the years 2007–2020. It is seen that developed countries have experienced worse in comparison with developing ones. FDI inflows have been suffered by the impact of COVID-19, mostly on investments in global value chance (GVC)-intensive, tourism, and resource-based activities in developing and transition economies [59]. On the other hand, greenfield FDI inflows had been facing a reduction since 2018. This reduction became steeper with the pandemic, particularly in developing countries. Africa is the most affected region in greenfield FDI flows with a 65% decline. Latin America and the Caribbean hold second place with a 51% decline, and it is followed by Asia [8].

Global FDI outflows have been weathered the same way amid in COVID-19 outbreak. This unfavorable impact of the health crisis has been expected to be intense in developing countries. The negative effect is uneven because the majority of FDI flows to developing countries are based on the primary sector. In other words, FDI flows in those countries are focused on commodities, where prices have fallen down by lack of demand as a result of the pandemic restrictions [44, 58]. This has led to FDI outflows in emerging countries, and according to the IMF, between the beginning of the pandemic outbreak and March 2020, approximately $83 billion have drawn back from those countries. That was the largest FDI outflow the world has ever experienced [31].

**Fig. 2:**
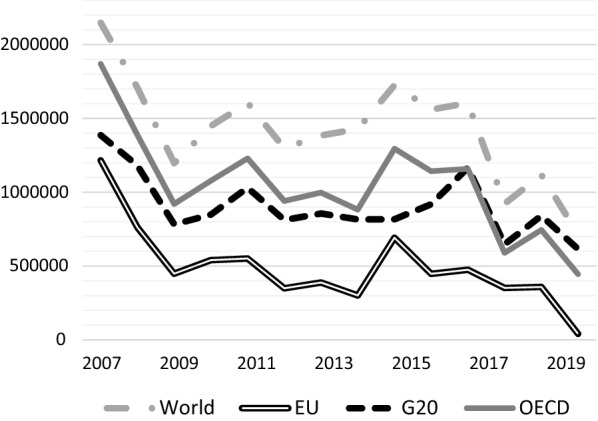
FDI outflows by selected areas (Million dollars). *Source*: OECD (Authors’ Compilation)

**Fig. 3:**
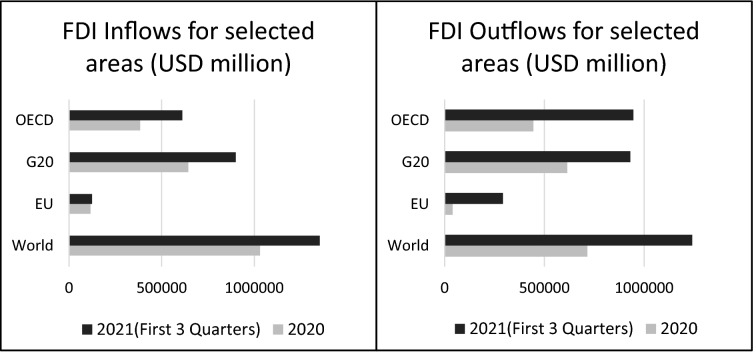
FDI inflows and outflows by selected countries. *Source*: OECD (Authors’ Compilation)

Figure 2 shows FDI outflows from selected areas between the years 2007 and 2020. The downward path of FDI outflows in the first year of the pandemic can be clearly observed. The European Union (EU) has experienced the biggest shrinkage in FDI outflows with the rate of 88%. With the beginning of the vaccination, the pessimistic atmosphere has turned into a good one by the first months of 2021. Global FDI flows increased to 870 billion dollars surpassing pre-pandemic levels and this increase was 43% greater than in the first half of 2019. Countries with the highest FDI flows in this period were the USA, China, and the UK with a more than USD 20 billion rise [47]. On the other hand, in Fig. 3, the tendency of FDI inflows (left side) and outflows (right side) for the year 2020 and the first 3 quarters of 2021 are given. In developed countries, new investment movements have enhanced somewhat thanks to the projects in the healthcare and manufacturing sectors. However, the greenfield investment projects maintained their decrease in many emerging markets and developing economies [47].

## Related literature

The novel COVID-19 outbreak is not the first pandemic that has been witnessed. Spanish flu in 1918, Asian flu in 1957, H1N1 Swine flu in 2009, West African Ebola in 2014, and Zika virus are some examples of pandemics and epidemics the globe had seen before. Increasing diseases and their ambiguous consequences are generated big challenges both on regional and global economies. For this reason, the socioeconomic impacts of pandemics are intensively examined by researchers.

Garrett [27] has investigated the effects of the Spanish flu via the compiled print media evidence due to the absence of economic data back in those days. One should remind that the Spanish Flu emerged in the last months of World War I. Therefore, both war and the pandemic have caused many deaths, notably, households’ primary breadwinners' deaths. This situation had unfavorable influences on consumers' confidence, behavior, saving, investment, income growth, long-run human capital, aggregate output, and production [27]. Almond [5] has evaluated the effects of the pandemic from a different perspective. The author has considered the fetal origins hypothesis, which suggests that there is a positive correlation between humans' health endowment and their abilities, productivity, and thus wages and revenue. For this, Almond [5] employs 1960–1980 Decennial Census Data to examine the grownup results who born in America around the time of the 1918 Pandemic. Evidence of the study shows that people who were in utero during the 1918 pandemic have reached lower education levels, higher disability rates, and lower incomes.

Chou et al. [22] have investigated the possible effects of SARS outbreak in 2003 on several industries, and according to their results, tourism is the most affected sector by disease in comparison with other industries. The tourism sector is very delicate to external shocks, and because of its nature, the virus is more likely to spread by the industry [55], on the other hand, have determined that SARS had a harsh impact on the demand, local consumption, export, and air travel. Another disease with severe socioeconomic effects is caused by the Ebola virus. Its worst impact was on food security. It has only not reduced food, it also adversely affected people's eating habits. People had to alter their consumption habits and eat less than before the virus. Having access to education and health services has become more difficult with the Ebola, besides, it has significantly hindered the capacity of the countries to accomplish their poverty reduction goals [60]. The impacts of COVID-19, which has been affecting the whole world for 2 years, on the general economic indicators such as unemployment, agricultural production, growth, trade, and tourism have been investigated by several researchers [15, 35, 39, 56, 61] However, since the role of the COVID-19 outbreak on FDI is the main object of this study a summary of the studies specifically on the FDI is given.

 [26] have presented the answer of how the COVID-19 pandemic affected home and host countries’ FDI margins for 96 countries using the data set of January 2019 to June 2020. Two measurements of each country, namely new COVID-19 cases and new deaths caused by COVID-19 have been chosen to analyze the effect of pandemic data on FDI announcements, FDI rumors, and FDI completion, separately. The results for Heckman estimation showed that home countries’ mortality rates for COVID-19 diminished extensive FDI margin and also, host countries’ pandemic situation for both OECD and especially emerging countries is a distinctive feature of the sensitiveness of FDI. Lastly, they reported that the most severely affected FDI sector is the service sector. [24], in a study for 43 countries, including OECD countries, BRICS countries, and Singapore between 2009Q1 and 2020Q3, investigated the effect of COVID-19 on China’s FDI inflows. For the analysis, they have focused on five different proxies, such as new cases, new deaths, cumulative cases, cumulative deaths, and active cases, for the COVID-19 pandemic. The regression results indicate that the numbers of new confirmed cases, new deaths, and cumulative confirmed cases have significant deleterious effects on FDI, with an average elasticity of around 0.7%. Also, empirical evidence shows that the impact of the industrial production index on FDI is significant.

In their recent paper, [43] have examined the relationship between the COVID-19 pandemic and FDI for 79 developed and developing countries. They have also argued how sovereign wealth funds affect the COVID-19–FDI nexus for countries with sovereign wealth funds and countries without sovereign wealth funds using a cross-sectional ordinary least square. The results of the study have revealed the negative correlation of foreign direct investment with both the number of death and the total cases from COVID-19. Another important finding suggests that the COVID-19 pandemic diminished significantly foreign direct investment in countries without sovereign wealth funds, while the effect is insignificant in countries with sovereign wealth funds. [29], in a panel data study on a larger data set of 142 economies and sub-samples (incomes and regions), perused the effects of the sudden outbreak of COVID-19 on FDI. The sample period covers the period from 1996 to 2019. In this study, unlike the data used in other pandemic studies, the new world pandemic uncertainty index was utilized for COVID-19. Growth, domestic investment, human capital, financial development, environmental factor, energy security, and lastly trade openness were used as control variables in the analysis. The results for the two-step system generalized method of moments estimation of the linear dynamic panel data model (DPDGMM) show the negative effects of health pandemics on FDI. In the study, it was also reported that the uncertainty stemmed from pandemics builds adverse shocks net inflows in FDI in both Asia–Pacific countries and emerging economies.

Giofré [28] have examined trends in foreign investment immediately after the adoption of stringent government measures to contain the spread of COVID-19 by using the Stringency Index (SI) as an indicator of the stringency of containment measures taken, and data on new COVID cases and deaths for 53 countries. Evidence of the robust least squares, standard OLS, and quantile regressions show that foreign portfolio investment, which tends to be more volatile and reactive than foreign direct investment, responds more strongly to immediate government intervention than to gradual intervention at the end of the first quarter, suggesting that the former policies are perceived as a more serious commitment to curbing the spread of COVID-19. By contrast, in the second quarter, the standard deviation of the index captures the abrupt withdrawal of containment policies along with the timely adoption of measures and loses significance for foreign investors.

## Data set and methodology

In this section, the data set is introduced and then the econometric methodology that is used in the empirical analysis is discussed.

### Data set

The study explores the effects of the COVID-19 outbreak on foreign direct investment using the world pandemic uncertainty index of 12 emerging countries (Brazil, Chile, Colombia, Czech Republic, Hungary, India, Indonesia, Mexico, Poland, Russia, South Africa, and Turkey) over the period of 2014Q1–2021Q3. The role of uncertainties on economic manners has intensively been investigated in the literature [10, 12, 21, 32].^2^ Ahir et al. [2] have generated a new uncertainty index, named *World Uncertainty Index (WUI)*. This index reflects all events such as disease outbreaks, wars, financial and debt crises attacks, trade restrictions, referendums (Brexit, etc.), and the US presidential elections [2]. Due to the speeding up of COVID-19 pandemic, the World Pandemic Uncertainty Index (WPUI) was constructed at the global and country levels in 2020 by Ahir et al. [2]. With the index, it is aimed to catch up uncertainties of the global pandemics namely SARS, Avian flu (H5N1), Swine flu (H1N1), Middle East respiratory syndrome (MERS), Bird flu, Ebola, COVID-19, and Influenza (H1V1) [29]. There are limited papers that have investigated the economic impacts of the pandemic uncertainty index [6, 23, 52]. Besides, there is only one study [29] that has focused on the relationship between pandemic uncertainty and foreign investment, where the main motivation of this study was triggered. Ho and Gan [29]'s data spans from 1996 to 2019, but the intense effects of COVID-19 have occurred after 2019. Thereby, the authors believe that the data set of the current study will more accurately reflect the real impacts of the COVID-19.

According to the literature, it is expected that pandemic uncertainty has an inverse influence on net foreign direct inflows. Apart from this, gross domestic product and the exchange rate are added to the model as control variables. It is expected that the relationship between FDI and GDP is positive. GDP is an important macroeconomic variable as it gives information about the size and performance of an economy. An increase in real GDP is interpreted as a sign that the economy is doing well. Therefore, this situation creates an optimistic perception for investors and economic agents as the economy of a particular country is in good condition [4, 19]. On the other hand, the impact of the exchange rate on FDI is controversial. A real depreciation of a given country's currency increases the relative wealth of foreign investors and thus, foreign direct investment inflows in that country tend to increase [25]. There is, however, another assumption that there might be a negative relationship between exchange rate and exchange rate uncertainty and FDI inflows due to uncertainties arising from the exchange rate. The uncertainties about the potential returns of foreign investments in receiving countries deter firms to make an investment [53].

Table 1 presents the variables, their definitions, and the source where the data have been obtained. Gross domestic product is seasonal adjusted. The natural logarithm is taken for the gross domestic product and exchange rates. Since foreign investments comprise negative values and the pandemic index contains zero values, raw data are used in the study. In panel regression analysis, it is essential to add more control variables to avoid omitted variables bias. But having accessibility to data has been decisive in the selection of the countries and variables. The difficulty of accessing monthly and quarterly data for developing countries prevent doing a comprehensive study for those ones. It is therefore important to note that this study includes the largest possible number of countries with the longest period for the variables analyzed, considering the availability of data. The descriptive statistics, including mean, median, minimum, and maximum values of the data are reported in Table 2 to understand the statistical properties of foreign direct inflows, world pandemic uncertainty index, real GDP, and the exchange rate. As seen from Table 2, foreign direct investment has the lowest minimum value with − 16,931.69 followed by pandemic uncertainty index. In addition, the maximum value of the pandemic index is just under 68. The distribution of variables is explored by using Shapiro–Wilk [54] normality test and it is determined that the series are not normally distributed. The following model is used to examine the impact of the COVID-19 pandemic on foreign direct investments:

1 $${\text{FDI}}_{it} = f({\text{WPUI}}_{it} ,{\text{GDP}}_{it} ,{\text{ EXC}}_{it} )$$

**Table 1:** Variable definition

Variable	Definition	Database
FDI	Foreign direct investments net inflow (Million US dollars)	OECD [48] https://data.oecd.org
WPUI	World pandemic uncertainty index	Ahir et al. [2] and WPUI [63]
GDP	Gross domestic product (2015 = 100) (Million US dollars)	OECD [48] https://data.oecd.org
EXC	Exchange rates	OECD [48] https://data.oecd.org

**Table 2:** Descriptive statistics

	Mean	SD	Min	Max	Shapiro–Wilk Test
*FDI*					
Overall	5135.9	5414.01	− 16,931.69	38,167.48	0.88***
Between		4091.19	384.34	13,929.87	
Within		3731.91	− 12,170	41,209.11	
*WPUI*					
Overall	3.27	9.65	0	67.56	0.39***
Between		1.70	0.95	5.71	
Within		9.52	− 2.44	65.99	
*LGDP*					
Overall	14.1	1.01	12.43	16.04	0.94***
Between		1.05	12.56	15.89	
Within		0.07	13.87	14.29	
*LEXC*					
Overall	4.22	2.59	0.75	9.62	0.91***
Between		2.70	1.29	9.52	
Within		0.18	3.55	4.95	

*L* denotes logarithmic transformation, *FDI* Foreign direct investment, *WPUI* World pandemic uncertainty index, *GDP* Gross domestic product, and *EXC* Exchange rate. Shapiro–Wilk Hypothesis Test for H_0_: The variables are normally distributed

***Indicates that the Null hypothesis is rejected at 1% significance level

### Methodology

In this section, we present the information about the panel quantile regression analysis.

### Panel quantile regression

This paper employs a panel quantile regression model that accounts for unobserved heterogeneity and heterogeneous covariates to broadly examine the impact of the COVID-19 outbreak on foreign direct investment. By applying a panel quantile regression method, one can obtain the estimates of the different slope parameters at different quantiles of the dependent variable [64]. In this way, we can analyze the determinants of FDI through conditional distribution, particularly in the countries with the most and the least FDI.

The extensively used ordinary least squares (OLS) method is an estimation technique in which all weights of observations, including outliers, are equal. However, the OLS method focuses on the mean effects. OLS estimators also lose their efficiency, especially in cases where the errors are not normally distributed, and the mean is affected by equally weighted outliers. The quantile regression model developed by [37] does not adhere to a conditional assumption about the distribution of the data, such as the normal distribution. However, it has become a frequently preferred method because of numerous advantages over the limitations of the OLS method. Subsequently, the quantile regression model was adapted to the fixed-effects panel quantile data approach by [36]. The panel quantile regression, based on estimating the conditional median of the dependent variable, allows for different weights for different values in the conditional distribution. Thus, the regression makes it possible to estimate the behavior of each specific point in the conditional distribution [3]. In this way, the method ensures that both more robust estimators are obtained and the effects of outliers on the results are eliminated.

The general quantile conditional function for quantile $\tau$ regression can be defined as in Eq. 2 [9]:

2 $$Q_{{{\text{FDI}}_{it} }} (\tau |\gamma_{i} ,\delta_{t} ,X_{i,t) } = \gamma_{i} + \delta_{t} + \alpha_{1,\tau } {\text{WPUI}}_{i,t} + \alpha_{2,\tau } {\text{LGDP}}_{i,t} + \alpha_{3,\tau } {\text{LEXC}}_{i,t} + u_{it}$$

where $\tau$ represents quantiles, including 25th, 50th, and lastly 75th, *i* is for cross sections, *t* for the time-period, and FDI is dependent variable.

More importantly, in this study, we also used quantile regression with correlated random effects (CRE) following [1, 11]. The motivation for using the CRE quantile regression methods is that the model does not suffer from an incidental parameters problem stemming from the inclusion of a considerable number of fixed effects and performs well even when omitted effects have scale effects on the response.^3^ This methodology overcomes the difficulties of fixed-effects models in short panels and provides results based on more information about the cross section on random effects.

Our econometric methodology includes the following steps: cross-sectional dependence, slope homogeneity tests, unit root tests, and finally quantile panel regression.

## Results and discussion

The starting point, and the most important part of panel data analysis, is to check the existence of cross-sectional dependence (CD) stated as cross-sectional interaction in the model. The reason is that the econometric techniques used from the first to the last stage of the analysis may differ depending on whether there is cross-sectional dependence in the panel data set. Thus, the key problem is to detect the presence of cross-sectional dependence in the model. The unit root test to be applied in the next step depends on whether there is cross-sectional dependence in the model. The first-generation panel tests do not account for cross-sectional dependence, whereas the second-generation panel tests compensate for the lack of cross-sectional dependence in the first-generation tests. Table 3 reports the results of four cross-sectional dependence tests including [17] Lagrange multiplier (LM), CD and scaled LM by [50], and bias-corrected LM (LM_adj_) by [51].

**Table 3:** Cross-sectional dependence results

Model	LM	CD	CD_LM_	LM_adj_
${\text{FDI}} = f\left( {{\text{WPUI}},{\text{LGDP}},{\text{LEXC}}} \right)$	84.19* (0.07)	− 0.92 (0.36)	1.58 (0.11)	1.38 (0.17)

L denotes logarithmic transformation. The numbers in parentheses are probability values

*Indicates that the Null hypothesis is rejected at 10% significance level

The null hypothesis of no cross-sectional dependence cannot be rejected at the 5% level for the test statistics in Table 3, except for the statistic LM. The statistics of LM cannot clearly confirm the alternative hypothesis of cross-sectional dependence, while the other statistics accept the null hypothesis. Based on these test statistics, it can be said that there is no dependence between the cross sections, in other words, the countries are not affected by the positive or negative shocks of the other countries. One of the possible reasons for the inability to detect cross-sectional dependence between countries could be that the sample consists of 12 countries. Following the results of cross-sectional dependence, the results of the slope homogeneity test developed by [51] are presented in Table 4. The calculated statistics of Δ and Δ_adj_ reject the null hypothesis that the slope coefficients are homogeneous and accept the alternative of slope heterogeneity for the model. These results provide sufficient support for the use of quantile regression for further analysis.

**Table 4:** Slope homogeneity test

Δ	Δ_adj_
− 1.79* (0.07)	− 196** (0.05)

The numbers in parentheses are probability values

*And ** indicate that the Null hypothesis is rejected at 10% and 5% significance level, respectively. H_0_: slope coefficients are homogeneous

Determining the unit root properties of variables is as important in panel data analysis as it is in time series analysis. Due to a consequence of the lack of cross-sectional dependence across countries, and the presence of a heterogeneous panel data set confirmed by the slope homogeneity test, we next apply the first-generation panel unit root test to determine the stationary properties of the variables. For this purpose, the Levin–Lin–Chu panel unit root test [38] (LLC) and the Im–Pesaran–Shin panel unit root test [33] (IPS), which are the most popular among the first-generation tests, are preferred. The results of the panel unit root test are illustrated in Table 5.

**Table 5:** Panel unit root test results

	Level	
	LLC	IPS
FDI	− 12.29*** (0.00)	− 12. 46*** (0.00)
WPUI	− 7.26*** (0.00)	− 6.80*** (0.00)
LGDP	− 2.81*** (0.00)	− 1.40* (0.07)
LEXC	− 4.03*** (0.00)	− 2.63*** (0.00)

L denotes logarithmic transformation. The numbers in parentheses are probability values

*And *** indicates that the Null hypothesis is rejected at 10% and 1% significance level, respectively. Lag length has been chosen 1 according to the AIC

The results of both the LLC and IPS unit root tests, which do not account for cross-sectional dependence, show that the FDI, WPUI, GDP, and LEXC series are stationary at the level. The null hypothesis of unit root for all series at their levels is rejected at the 1% statistical significance level for both tests. The empirical evidence from the unit root tests indicates that all series have no unit root at their levels, thus they are all I(0).

This study addresses the question of whether explanatory variables have differential effects on the conditional quantiles of foreign direct investment using panel quantile regression models. For this purpose, three estimation methods, including the Mean model, the fixed-effects panel quantile regression (FE), and correlated random effects (CRE) quantile regression, are taken into consideration in order to obtain more reliable and comparable results. Especially the FE panel quantile regression enables us to specify the impacts of WPUI and other control variables whether differ based on the level of FDI inflows. Further, the CRE panel quantile regression performs better than FE using information constructed from all observations for each individual [11]. The empirical outcomes of the Mean model, the fixed-effects panel quantile regression, and correlated random effects quantile regression are given in Table 6.

**Table 6:** Panel quantile regression results

	Mean	FE			CRE		
		Quantiles			Quantiles		
		0.25	0.50	0.75	0.25	0.50	0.75
WPUI	2.78 (0.13)	− 2.32** (− 2.13)	− 2.35* (− 1.79)	− 3.04 (− 0.85)	− 2.32** (− 2.22)	− 2.35** (− 2.01)	− 3.04 (− 1.00)
LGDP	9850.37*** (3.22)	1.98*** (5.47)	2.22*** (7.55)	2.55*** (6.09)	1.98*** (5.18)	2.21*** (6.87)	2.55*** (5.65)
LEXC	− 35.17*** (− 2.90)	4.29 (0.36)	2.74 (0.24)	− 7.40 (− 0.47)	4.29 (0.32)	2.74 (0.22)	− 7.40 (− 0.50)
Number of observation	31						
F-statistic	4.59***						

L denotes logarithmic transformation. The numbers in parentheses are t statistics

*, ** and *** indicate that the Null hypothesis is rejected at 10%, 5%, and 1% significance level, respectively. FE: Fixed-effects panel quantile regression, CRE: Correlated random effects quantile regression

According to the Mean results, the impact of WPUI on foreign direct investment is positive but insignificant at the 10% level. As known, the result of the Mean model is not sufficient to demonstrate the impact of the COVID-19 pandemic on foreign direct investment. On the other hand, the coefficient of GDP is highly significant and has a positive sign. Regarding the exchange rate, the impact of the EXC on foreign direct investment is negative and significant at the 1% level. Note, however, that these conventional regression coefficients may be under-estimated or over-estimated because the methodology focuses on the mean effects [16].

The motivation for the quantile approach is that it accounts for heterogeneity in the distribution to provide a detailed analysis of the relationship of world pandemic uncertainty index, GDP, and the exchange rate as independent variables across the different quantiles of foreign direct investment as dependent variables. In this study, we run the quantile regression for the selected quantiles of the 25th quantile, the 50th quantile, and the 75th quantile, which are, respectively, the percentiles of the conditional FDI distribution for low-, medium-, and high-foreign direct investment in host countries. So, the statistics obtained here show the impact estimates of the world pandemic uncertainty index and other control variables to FDI at low, medium, and high quantile levels. Hübler [30] states that the different quantiles of the 50th represent the median. According to the literature, a negative relationship between the COVID-19 pandemic and foreign direct investments should be expected.

As can be seen in Table 6, the findings obtained from each panel quantile regression model differ substantially from the Mean test results in the study. The FE and CRE panel quantile regression results show that the impacts of the COVID-19 pandemic, GDP, and the exchange rate on FDI are generally very heterogeneous. Moreover, the results are also quite distinctive between the quantiles. According to FE and CRE panel quantile regression models, it can be said that the impact of the COVID-19 pandemic on foreign direct investment is clearly heterogeneous. The coefficients are negative, as expected, and become significant at the lowest quantile (0.25th quantile) and the middle quantile (0.50th quantile), implying that the influence of the COVID-19 pandemic on FDI is detrimental, and the effects are more significant for the low-FDI countries and the middle-FDI countries than the highest quantile. The coefficient is negative but insignificant at the highest quantile (0.75th quantile).

The negative coefficient of the COVID-19 pandemic is statistically significant at the 5% level for the 0.25th quantile. The estimated coefficient of low-FDI countries shows that every one-unit increase in WPUI leads to a decrease in FDI by 2.32. This coefficient in the CRE panel quantile regression remained the same in terms of both the impact and significance with FE panel quantile regression. For the 0.50th quantile, the coefficient of the COVID-19 pandemic is estimated to be -2.35. This coefficient is statistically significant at the 10% level according to FE panel quantile regression. However, the coefficient obtained by the CRE method is statistically significant at the 5% level, which provides more reliable results. The results demonstrate that the unit effects of the COVID-19 pandemic on FDI are quite similar in the low-FDI countries and middle-FDI countries, however, the effect differs considerably in high-FDI countries, where the effect is negligible. As mentioned before the results are obtained as expected. Foreign direct investment inflows in those countries are mainly on the manufacturing sector, tourism, wholesale and retail, and gas and oil extraction. In addition, foreign investments have a significant share in the exports and GDP of the countries. During the pandemic, the demand had decreased due to restrictions, thereby the investments made in these areas have declined. Apart from this, the reasons such as macroeconomic instability, weak economic competitiveness, unpredictable regulatory policies, lack of transparency, and low company valuations of those countries make them more vulnerable to crises [13, 18, 57, 59].

At the 0.75th quantile, the detrimental impact (-3.04), of WPUI on FDI is higher than at the 0.25th and 0.50th quantiles, but the coefficient is not statistically significant at the 5% level. Thus, the impact of the COVID-19 pandemic on FDI is eliminated because it is not statistically significant in countries with high levels of FDI. The collapse in FDI inflows throughout emerging countries and regions was uneven. For instance, FDI inflows in India have risen because of M&A activity, unlike the inflows that have decreased elsewhere in the region [59]. Also, it is worth noticing that in the results of the Mean models, the impact of the COVID-19 pandemic on FDI is found to be statistically insignificant, whereas this impact is found to be negative and statistically significant for the 0.25th quantile and the 0.50th quantile in quantile regressions.

Empirical evidence shows that the devastating impact of the pandemic on the flow of foreign direct investment varies across emerging countries. The countries that felt the harmful impact of the pandemic on net FDI inflows the most are those with low-FDI and middle-FDI countries. Net FDI inflows decline significantly along with the surge of the impact of the COVID-19 pandemic, and the inverse impact is the highest in the lower and middle quantiles. The devastating impact of the pandemic on net FDI inflows is also confirmed by the studies of [24, 28, 29, 43].

GDP has a positive effect on FDI in both the FE and CRE panel quantile regression, as in the Mean model. The results of the quantile regressions show that the impact of GDP is higher in higher quantiles. The positive impact diminishes in lower quantiles. From the 0.25th quantile to the 0.75th quantile, the estimated coefficients of GDP are 1.98, 2.22, and 2.55, respectively, for the two-panel quantile regression approach. While a 1% increase in the GDP increases the FDI by 1.98% in the low-FDI countries, a 1% increase in the GDP increases the FDI by 2.55% in the high-FDI countries. The results for GDP imply that growth is a dominant factor for FDI inflows in all emerging economies, especially more dominant in high-FDI countries. Table 5 clearly shows that the rise in FDI due to GDP is prominent and soars at a higher magnitude at the 75th quantile. On the other hand, the impact of EXC on FDI is positive at the 25th quantile, at the 50th quantile, and is negative at the 75th quantile. But the impact of EXC on FDI is insignificant at all quantiles. Contrary to the Mean method, both the FE and CRE panel quantile regression results show that the exchange rate has no impact on net FDI inflows for lower FDI inflows countries.

## Conclusions

The whole world has been collapsed by the COVID-19 outbreak and the uncertainties it created. Governments around the globe implemented various measures to mitigate the spread of the virus such as shutdowns and travel restrictions. The pandemic caused a complete change in the habits of societies in areas such as policy decisions, health, daily life, and entertainment, which had devastating effects on the global economy. The current study gauged the role of the COVID-19 outbreak on the foreign direct investment inflows via panel data of 12 emerging economies from 2014 to 2021. In order to explore the impact of the pandemic on foreign investments, panel quantile regression has been employed which estimates the conditional median of the dependent variable and provides a more comprehensive analysis of the relationship between variables.

Findings show that the pandemic uncertainty index has different impacts at different levels of foreign direct investment and the negative impact of WPUI increases as the quantiles of FDI rise. Results are in line with prior expectations. FDI inflows in emerging economies have dropped because the primary and manufacturing sectors which constitute a large part of FDI inflows in those countries had seriously been touched by the pandemic. However, the adverse impact of the pandemic uncertainty index on foreign direct investment slightly differs from lower quantile (0.25) to the median (0.5). For the low- and middle- FDI countries, every one-unit increase in WPUI leads to a decrease in FDI by 2.32 and 2.35, respectively. On the other hand, GDP has a positive impact on foreign direct investment inflows and this positive effect diminishes in lower quantiles and rises in higher quantiles. Finally, the impact of the exchange rate on foreign investments is heterogeneous at distinct quantile levels but it is statistically insignificant at all quantiles.

Uncertainties highly affect international investors' attitudes. The pandemic crisis has far more disrupted foreign investment in emerging countries, which are weaker and more vulnerable in terms of the economic structure, than developed countries. Therefore, the economic structure of countries is an essential determinant in receiving investments. The principal shortcomings of emerging economies in attracting international investments are their unpredictable and non-transparent policy implementations. Therefore, policy decisions in these countries should primarily be purposed creating a more reliable investment environment that will help countries struggle in times of global crisis. On the other hand, climate change is cited as one of the reasons for health crises such as pandemics. This situation shows that the policy decisions of the countries should be toward attracting the right investments in the right areas. Therefore, emerging countries should encourage greenfield investments, which open new and broader economic opportunities for the host country. In addition, the investments in question should be following the sustainable development goals. To do so, new tax policies and incentives, new trade agreements, retraining of the workforce in line with the demands are among the crucial implementations. In addition, COVID-19 has ushered in new ways of both individuals and companies' behaviors all over the world, and therefore, more flexible decisions are required according to the rapidly changing global trends. Finally, the researchers should consider the sectors for studies focused on foreign direct investments. The effects of the pandemic might change from sector to sector. This will provide more comprehensive results to analyze the size of the effect of the pandemic between the sectors.

## Data Availability

The data used can be downloaded from WPU and OECD, respectively, using the following links: https://worlduncertaintyindex.com/data/ and https://data.oecd.org. The relevant codes will be made available upon acceptance as supplementary material.

## Abbreviations

APTIT: Asian-Pacific trade and investment trends
CD: Cross section dependency
CRE: Common random estimation
EU: European Union
EXC: Exchange rate
FDI: Foreign direct investment
FE: Fixed effect
GDP: Gross domestic product
IMF: International Monetary Fund
IPS: Im–Pesaran–Shin
LLC: Levin–Lin–Chu
LM: Lagrange multiplier
ODI: Overseas development institute
OECD: Organization for Economic Co-operation and Development
OLS: Ordinary least square
UNCTAD: United Nations conference on trade and development
UNDG: United Nations development group
WPUI: World pandemic uncertainty index
WUI: World uncertainty index

## Footnotes

1 The countries used in the study are Brazil, Chile, Colombia, Czech Republic, Hungary, India, Indonesia, Mexico, Poland, Russia, South Africa, and Turkey. According to [58] Brazil, India, Indonesia, Mexico, and Russia are among the top 20 FDI host economies.

2 For more information on the relationship between uncertainties and economic behaviors, see [7] “Economic policy uncertainty: A literature review.”.

3 For more detailed information on CRE quantile regression method, see [11] “Headlights on tobacco road to low birthweight outcomes.”.
